# Observations on Blindness

**Published:** 1954

**Authors:** R. Ramsay Garden

**Affiliations:** Senior Surgeon, Bristol Eye Hospital; Ophthalmic Surgeon, United Bristol Hospitals; Clinical Teacher in Ophthalmology, University of Bristol


### OBSERVATIONS ON BLINDNESS

### 'he Presidential Address, delivered on Wednesday, 14th October, 1953, t the opening of the Seventy-Fifth Session of the British Medico-Chirurgical Society

BY

R. RAMSAY GARDEN, M.A., M.B., CH.B., D.O.M.S.

*'enior Surgeon, Bristol Eye Hospital; Ophthalmic Surgeon, United Bristol Hospitals; Clinical Teacher in Ophthalmology, University of Bristol*

> " Why was the sight
> To such a tender ball as th' eye confin'd?
> So obvious and so easie to be quench't "
>
> MILTON: *Samson Agonistes*

When I came to consider possible themes for this address, it seemed to me that ›me observations on blindness would be an appropriate choice. The subject oes not appear to have been discussed at any length in this Society before, and ıy acquaintance with blind folk of all ages has been an extensive and fairly ıtimate one, during many years spent in clinical ophthalmology, in certifying ıe blind in this area, and, until recent years, in looking after the eyes of children : the Royal School for the Blind.

### COURAGE OF THE BLIND

I have been further influenced in this choice by a genuine admiration for the ›urageous and often philosophical attitude of the blind towards their affliction -found not only amongst the aged blind, in whom might be expected a meas-:e of acquiescence in their failing powers. Children born without sight are ı a separate category, for they have never known anything different, and in my ‹perience there is a distinctly cheerful, lively atmosphere in a modern school ›r the blind. The lot of those blinded in youth or in their working years is ırder, although they are greatly helped in their adjustment to sightless condi-›ons by a store of visual memories. Nevertheless, whatever the age, previous ›cation, or social background of the many hundreds of blind people I have met, am always amazed and cheered by the spirit in which the great majority of ıem accept the loss of sight. Possibly those most deprived and handicapped ʼ blindness are the highly educated, intellectually keen elderly people, who have ›pended for most of their enjoyment in life on reading, and who find difficulty acquiring fluency in the reading of Braille or Moon type. For them, however, ıe " Talking Book " has come as a godsend, and with an ever-expanding library goes far to make up for the deficiencies some people find in broadcasting. Comparative contentment in the blind state may depend on the sympathy engendered in those around; sometimes it may arise partly from a sense of security, for no group of the handicapped have been so well protected by legislation and voluntary effort.

Although some authors have written in the opposite sense, I doubt very much if there is any abnormal psychology of blindness, or any unusual type of personality produced by it. The reaction to blindness is determined, I believe, by the character of the individual, just as in other crises or tragedies in life. The poet Milton, blind from the age of forty-four, is said to have accepted his lot with courage and cheerfulness, and there is little of self-pity in any references he makes to his own blindness—as witness the sonnet addressed to his pupil and friend, Cyriack Skinner:

Cyriack, this three years day these eys, though clear
To outward view, of blemish or of spot;
Bereft of light thir seeing have forgot,
Nor to thir idle orbs doth sight appear
Of Sun or Moon or Starre throughout the year,
Or man or woman. Yet I argue not
Against heavns hand or will, nor bate a jot
Of heart or hope; but still bear up and steer
Right onward. What supports me, dost thou ask?
The conscience, Friend, to have lost them overply'd
In libertyes defence, my noble task,
Of which all Europe talks from side to side.
This thought might lead me through the world's vain mask
Content though blind, had I no better guide.

Milton's contentment in blindness, and in the face of his many domestic and other trials, was doubtless strengthened by the literary activities with which his days were crowded.

### SOCIAL WELFARE

The literature on blindness in all its aspects is very voluminous, and the social aspects of blindness, the growth of educational and welfare services for the blind, can only be outlined briefly, although their history is absorbing. In most European countries, the record of efforts by religious bodies and philanthropic individuals to relieve the sufferings and poverty of the sightless covers several centuries. Organized help for the blind at first took the form of asylums or hospices, the most famous of which was the Hôtel des Quinze-Vingts in Paris, still in existence today as an ophthalmic hospital and blind institution. This hospice was founded by Louis IX of France about the middle of the thirteenth century as a refuge, it is said, for three hundred of his Crusaders who, captured with him by the Saracens, were over a period of fifteen days blinded at the rate of twenty each day (hence " quinze-vingts ") as a barbaric method of hastening payment of the ransom demanded for the King's release.

Organized education for the blind, however, did not come about until 1784, when the first national school for blind children was established, also in Paris, by Valentin Haüy. His system of embossing paper with raised characters to provide reading-matter for the blind was later superseded by that of Braille (a pupil and later instructor at the school founded by Haüy), who developed and published in 1829 the six-point embossed type which is now used throughout the world for blind education of all kinds—literary, musical and scientific.

Following the example set in France, Liverpool opened a school for the blind in 1791, Bristol and Edinburgh in 1793, and London in 1799. From these beginnings, a comprehensive system of blind education, welfare and benefits has been evolved in this country, and it is in many respects the most complete in existence. It still incorporates a strong voluntary element—a combination of philanthropy and State financial assistance which has now practically disappeared from so many other national institutions. The activities of the largest of the voluntary bodies—the National Institute for the Blind—include the administration and maintenance of seven Sunshine Home Nursery Schools for blind infants, two secondary grammar schools, a school for blind children with other handicaps, a clinic and institute of physiotherapy, a training school for short-hand and telephony, and two residential rehabilitation centres for newly blinded adults. In addition there is its great publishing department, which prints embossed literature of all kinds (including text-books and scientific treatises for students) for blind persons of all ages.

Bristol's share in the education of blind children, in the training, employment and welfare of blind adults, and in the care of the aged blind, is a long and honourable one. The Royal School for the Blind, the Blind Asylum Workshops, and the three homes for old people, are institutions of which this City can be proud. It may be of interest to recall that the School was founded 160 years ago by two Quakers, Bath and Fox, who started with six pupils in an old meeting-house long since demolished during slum clearance. In 1803, it was moved to larger premises in Maudlin Street, on the site of the present Eye Hospital, and in 1838 to a building near the top of Park Street, where it remained until finally, in 1911, it came to rest in its present fine building in Henleaze Road. (The *Western Daily Press* of 4th February, 1953, contains an interesting account of the School and its development.)

The rehabilitation of the newly blinded is very important. Every year over 2,000 men and women between the ages of twenty-one and sixty-five years are added to the Register in England and Wales. They must not be allowed to brood and rust, but should at once be made to realize that they can become useful citizens again. They are usually in hospital or attending out-patients when the decision to recommend registration is taken, and through the Almoner and the local Blind Association application is made for admission to a Home of Recovery. There they learn to overcome difficulties in performing the usual tasks of daily life, to get about unaided, and gain facility in reading Braille, or in the case of older people the easier Moon type. Lessons in typewriting and handicrafts are given to most of the residents, and later on each individual's capabilities can be assessed. In the workshop, the sense of touch and manual dexterity can be developed by assembling parts, using a lathe, the Braille micrometer and other tools. Later, some may proceed for further training to Government centres, and some may perhaps return to take a useful share in their previous job, or other sighted industry. Two years ago, with a number of colleagues, I

spent a most interesting morning at Manor House, Torquay, one of the Queen Elizabeth Homes of Recovery for the Newly Blind. This house, and America Lodge (the gift of the British War Relief Society of the United States), are maintained by the National Institute for the Blind. There one finds people from all walks of life, spending varying periods of re-adjustment to new conditions, regaining confidence and hope, and eventually returning better fitted physically and mentally to take an active part in the life of the community.

The work of St. Dunstan's in the rehabilitation, training and re-settlement of blinded Servicemen is well known, but mention should be made of its activities and those of the National Institute in the invention and production of devices to help the blind in their work, recreation and locomotion. These range from writing machines, Braille watches and engineers' instruments, embossed playing-cards and games, to metronomes for musicians, and the "talking book" for those (and they are the majority) who have not mastered Braille or Moon type sufficiently to read embossed literature with ease and enjoyment. This invention, which I have seen, is a long-playing electric gramophone, adjusted to run at 24 revolutions per minute for British recordings, with an alternative speed of $33\frac{1}{3}$ r.p.m. so that American records can be played. It is so arranged that the users can do without sighted help, even when it comes to fitting a new pick-up. About 900 book titles are now available, chosen to suit various literary tastes, and are being added to at the rate of about fifty new recordings a year.

In regard to employment, the aim and tendency nowadays is to integrate the adult blind with the sighted as closely as possible. This is reflected in the increasing numbers of the blind who are employed in factories, and in a few new industries which are now within the capabilities of the blind.

### INCREASE IN BLINDNESS

Although we may well be satisfied with the progress of social welfare for the blind, there are other aspects which are not so pleasing, and some of them are important enough to deserve more detailed attention than could be given them in the general survey or progress-report I had originally contemplated. To begin with, the rising tide of the registered blind is disturbing, and since the problem of stemming it is by no means entirely an ophthalmological one, I thought this occasion might be an opportunity to place it before an audience comprising so many diverse sections of our profession.

Our methods of ascertainment and certification of the blind, if not yet entirely satisfactory, as will be explained later, are at least the equal of any used abroad. The document in use, familiar to the certifying ophthalmologist as B.D.8, is in process of being modified, mainly in the matter of etiological classification, which it is suggested should in future be left to a board of assessors to whom the clinical findings would be submitted. This would lead to uniformity, and might also be a step towards international agreement on nomenclature.

The definition of blindness in the adult and in the child is different—one being industrial, based on the individual's fitness for any sighted job, the other being wider and concerned with the impossibility of education by sighted methods. Some awkwardness occasionally arises when a child, leaving a school for the blind at the age of sixteen, is not regarded as blind industrially and has to take his chance, without training, in a sighted labour market. These considerations open up many questions, however, and need not be discussed now. What concerns us at the moment is the number of blind people certified under either definition.

Registration of the adult blind is not compulsory, but the statutory benefits which accrue have in the past ensured that a high percentage of those who come within the definition of blindness do actually apply for registration. A certain proportion of blind people, however, estimated at 10 to 15 per cent., remain outside the register—some because no one has suggested an application, others because the extent to which sight had failed had not been realized, and again because many do not appreciate that the standard of sight allowed by the definition and its interpretation is far higher than the word " blindness " usually connotes. I have even met people with exceedingly poor vision who had an illogical but in a way understandable reluctance to allow themselves to be labelled as blind in any sense. The idea would come to them as a surprise, and seemed the final inescapable blow to their cherished hopes of sight returning one day. On the other hand, I have known many patients with seriously failing sight who began, with great philosophy and courage, to equip themselves for a blind future by learning Braille long before there was any certainty of it being required.

When the Blind Persons Act came into force in 1920, the total of the registered blind was 30,785. It has now reached 86,389 and in the last two years for which figures are available (up to 31st March, 1952) 11,000 additions have been made annually. If we include an estimate of those who would be certifiable as blind but for some reason or another are not registered, the total would probably exceed 90,000. With the addition of another estimate of 10,000 for Scotland, the blind population of this island is around 100,000.

### CAUSES OF BLINDNESS

Several very detailed analyses have been published since 1938 based on large numbers of blind certificates completed in the United Kingdom. One of the more recent, by Sorsby (1950), deals with the causes of blindness in England and Wales as shown by certificates for the period 1933 to 1943. (Since my paper was read to the Society, the same author has published a further monograph on " The Causes of Blindness in England ", based on certificates for the years 1948 to 1950. Like its predecessor, it contains many interesting facts, figures and observations, which should be studied by all concerned with the prevention of blindness.)

In 1944, jointly with Professor R. H. Parry, I reported to the Bristol City Council on the 782 cases then on our local Register. Bristol being a city with a population so varied in vocations, and with such a variety of industries, there is within its boundaries a fairly representative cross-section of the English people. Although comparatively small in number, the Blind Register in Bristol (now containing 935 names) forms in a sense a microcosm of the country's blind population.

Between 1944 and the end of last year, i.e. in eight years, 622 new applicants were registered as blind. The individuals in both this and the other Bristol group of 782 previously mentioned were, with few exceptions, examined by myself, and it is upon experience gained in this connection, and in the cours of hospital and private practice, that my personal opinions are based.

It has been reckoned that in about 25 per cent. of the blind population th cause dates from infancy, and in over half of all blind people, the cause of ultimat blindness develops after the age of fifty years. In the Bristol blind populatio now numbered at 935, nearly half are over seventy years of age, and the Nationa Statistics for the year to 31st March, 1952 (the last available), show the greate increase in the 70 + age group. It is therefore obvious that the increasin number of the blind is becoming a geriatric problem of some importance. Mor people are living to the advanced years when senile changes causing blindne are to be expected, and as the average expectation of life is longer, many wh lost their sight in earlier years may survive to increase the numbers of the ag blind.

There is a good deal of evidence to show that towards the end of the eighteent century the commonest cause of blindness was smallpox. Vaccination and oth preventive measures steadily reduced the incidence of this scourge and i associated loss of sight, until by the end of the nineteenth century it had cease to be important in this respect. As one menace to sight went, another becam prominent—in the form of ophthalmia neonatorum. This by the end of th nineteenth and beginning of the present century had become responsible f about 40 per cent. of the admissions to the blind schools of this country. As i very well known, ophthalmia neonatorum has now almost disappeared, and f a baby to become blind in even one eye from this cause has become a gre rarity. This has been an outstanding triumph for preventive medicine. Credé method of treating the eyes of the new-born, compulsory notification of co junctival discharge within twenty-one days of birth, ante-natal treatment o gonococcal and other infections in the mother, with early and vigorous treatme of the baby's eyes when found to be infected—all these combined to reduce th rate and effects of the disease. With sulphonamides, it became possible to cur a case of ophthalmia in a matter of days instead of weeks and, with penicilli in a matter of hours.

Thus two of the major causes of blindness have been eradicated, and we hav now to consider the present-day situation. The frequency with which one fin cataract, glaucoma, the group of congenital, hereditary and developmenta anomalies, and high degrees of myopia, is quite striking. They account for least half of the total, and although the picture (in Bristol at any rate) is changin in at least one respect—the increasing incidence of blindness due to diabetes all the surveys studied show a preponderance of the principal causes mentioned

In the official B.D.8 form, the causes of eye defects leading to blindness a classified in four groups, excluding the one called " No information available It will be convenient, I think, to discuss the present causes of blindness withi the framework of these four groups, and to follow up with some observations o a few important advances in their prevention and treatment. The categories t be considered are the congenital and undetermined causes, the infectious an bacterial, the traumatic and chemical, and the effects of general disease.

*Congenital and Undetermined Causes*

This includes conditions such as congenital, hereditary and developmenta defects, myopia, cataract, glaucoma, and retinal detachment. In the first Bristol series, nearly 56 per cent. were included in this category and, in the second, 62 per cent. Although a large volume of research has been going on for many years, we know comparatively little that is entirely reliable about the basic causes of this, the largest group.

There is still much uncertainty about the etiology of the sporadic ocular anomalies present at birth. One often finds instances of normal individuals in families affected by what are generally regarded as hereditary or familial eye diseases, and it has also to be recognized that congenital and other ocular defects caused by intra-uterine toxins may be clinically identical with the hereditary types of anomaly. More will be said on this subject when we come to discuss the second group of causes. We are thus not on very sure ground when it comes to the controversial subject of eugenic control of marriage and parenthood. It has been stated that the effects of its application in reducing blindness from hereditary defects might well be counterbalanced on the whole by the loss of potentially useful citizens.

The ocular defects arising in infancy have been added to in an alarming fashion in recent years by the disease of prematurity known as retrolental fibroplasia, which has already accounted for many cases of blindness in babies. To the intensive research being carried on in various parts of the world on this subject, a notable contribution has been made in regard to its preventive treatment by our own colleagues, C. A. Brown and Beryl Corner (1952).

When we come to consider the eye diseases of uncertain origin in older people, we find that it is not yet known why senile cataract develops, or how one can prevent the structural and other changes associated with that most insidious and treacherous of all eye diseases, glaucoma. Many facts are available about the biochemistry and pathology of cataract—we know what happens, but not much of why it happens. On the other hand, we can restore vision by the old or new methods of cataract extraction, prevent serious loss of sight in many cases of early glaucoma, and preserve what remains in those where deterioration is already considerable.

Cataract and glaucoma are obviously the most common causes of blindness in this country, and when it comes to the possibility of remedial treatment, the patients in whom improvement of sight is feasible are, with a few important exceptions, those with cataract.

### *Infectious and Bacterial*

This group includes the results of venereal disease, tuberculosis, specific fevers, septicaemia and meningitis. The importance of interstitial keratitis and other eye diseases of specific etiology has declined to a noteworthy extent, for some of the reasons which operated in the case of ophthalmia neonatorum, and there has been a steady fall in blindness due to phlyctenular ophthalmia and the infections of childhood. This again is largely to the credit of public health departments which during several generations have done so much, on the one hand to improve the nutrition and hygiene of young children and, on the other, to lessen the risks to sight from infectious diseases by organizing prophylactic inoculations and by maintaining a high standard of treatment in isolation hospitals.

It was mentioned in the last section that intra-uterine influences might dete[illegible] mine the occurrence of congenital anomalies, as in toxoplasmosis, for exampl[illegible] Again, Gregg's observation in 1941, that congenital cataract was frequent [illegible] babies born of mothers who had contracted rubella in the early months of pre[illegible] nancy, created great interest at the time, and it is possible that other chorio[illegible] passing and placenta-passing viruses, toxins and bacteria may also have simil[illegible] ill-effects.

On the whole, it appears that the tendency has been for blindness due to i[illegible] fection and bacterial causes to decline, especially in children.

*Traumatic and Chemical*

Most of the injuries causing bilateral blindness in the relatively small numb[illegible] of cases in Bristol and elsewhere were non-industrial. Much *monocular* blin[illegible] ness is caused through industrial accidents, but for two eyes to be lost or for [illegible] second to be blinded by injury at work is not at all common. This, howev[illegible] does not justify any complacency about safety measures in factories or in t[illegible] home and streets, especially where children are concerned. I refer, of cour[illegible] to fireworks, darts, catapults, air-guns and other dangerous toys—the frequ[illegible] causes of serious injury to the sight of youngsters.

*General Diseases*

Eyes affected by general diseases are not usually amenable to treatment, a[illegible] any reduction in this sort of blindness will presumably depend on research i[illegible] the diseases responsible.

One great disappointment has been in diabetes, about which such high hop[illegible] were kindled when insulin was discovered. In spite of the strictest control [illegible] diabetes by insulin and associated treatments, retinopathy in young diabet[illegible] may show itself in ten to fifteen years, and in older patients within five and [illegible] years after onset of the disease.

At the Oxford Ophthalmological Congress in 1946, Professor Ballantyne end[illegible] the Doyne Memorial Lecture on " The State of the Retina in Diabetes " w[illegible] these words: " The physician has reason to be happy in that having establish[illegible] the diagnosis of diabetes mellitus he can in the vast majority of his cases ren[illegible] his patient 'sugar free' and fit to enjoy his normal activities with an almost norm[illegible] expectation of life. We must apologize if we disturb his peace of mind, but i[illegible] surely important to realize that over 30 per cent. of his patients have, or [illegible] acquire, pathological changes in the retina (not to mention cataract and oth[illegible] ocular manifestations of the disease), and that these pathological conditions [illegible] persist, and possibly advance to a stage at which they are a menace to visi[illegible] Unfortunately the apparently successful treatment of diabetes has no eff[illegible] whatever in preventing or arresting the march of the retinal disease—the fa[illegible] are a challenge, especially to ophthalmology, to probe more deeply into t[illegible] problems of this mysterious disease."

For me at any rate, those words have gained added force during the past f[illegible] years. An impression that I was seeing more diabetics as out- and in-patien[illegible] and that I was certifying more of them as blind, especially the older ones, [illegible] me to go into the figures in detail—with the following striking results: In [illegible] 1944 Bristol survey, diabetes accounted for only 0·5 per cent. cases of blindne[illegible]

in Sorsby's 1950 analysis for 1·8 per cent., but in the Bristol registrations since 1944 the proportion had risen to 5·9 per cent.

In the course of a discussion on "Ocular Complications of Diabetes" held at the British Medical Association Annual Meeting this summer, a number of interesting points emerged. According to G. I. Scott, in those seen within a year of the general disease being diagnosed, the incidence of retinopathy is probably 10 per cent., or less, while in patients who have had diabetes for twenty years or more the incidence is over 70 per cent. Diabetic retinopathy appears to be a distinct entity which can occur independently of hypertension; its incidence increases with the duration of the disease, and is not related to the severity of the diabetes. L. H. Howells, at the same meeting, emphasized that diabetes is becoming more common for a number of reasons—it is a hereditary disease; modern therapy increases fertility, makes childbirth safer, and improves the foetal survival rate in diabetic pregnancies. Moreover, diabetics now have an increased expectation of life, more of them live to develop the various ocular complications, and, as in about 30 per cent. retinopathy of some degree eventually occurs, "its prevention is the most important and challenging problem in the management of diabetes." (The opening papers read at the discussion by these authors have since been published in the *British Journal of Ophthalmology* for December.)

There is no evidence that senile cataract is commoner in diabetics than in non-diabetics of the same age, that senile cataract is accelerated by glycosuria, or that any kind of cataract is inhibited by insulin—or for that matter by any form of medical treatment whatsoever.

The necessity for continuous research in diabetes needs no emphasis, and it is to be hoped that before long the unknown factor concerned in producing ocular lesions, especially retinopathy, will come to light.

### ADVANCES IN TREATMENT

In the discussion so far, due credit has been given to State Medicine, and little said of the great part ophthalmology plays in the prevention and cure of blindness. Perhaps that is sufficiently obvious, but it is salutary in a profession like ours to pause now and then, to take a backward glance and re-assure ourselves that true progress is being made, to take stock of our present position and see what remains to be done in the future.

### *Medical Progress*

The efficacy of sulphonamides, penicillin and streptomycin, in certain bacterial eye infections is now well established. The value of the newer antibiotics in those eye diseases known or suspected to arise from virus infections, is at present under critical examination. So far the reports are only moderately encouraging, except in the treatment of trachoma with aureomycin, but it is probably only a matter of time before a useful therapeutic agent is discovered to prevent loss of sight from such viral conditions as dendritic corneal ulcer, herpes ophthalmicus and disciform keratitis.

Of all recent discoveries in the treatment of serious eye disease, ACTH and cortisone easily provide the most dramatic results. The use of these hormones in the treatment of general diseases will of course be well known to you all, but what the ophthalmologist is so grateful for is something which will be effective in preventing adhesions from blocking the pupil and exudates from clouding the cornea during the course of irido-cyclitis. One used to feel almost helpless at times when treating diseases of the uveal tract, and especially on the occasions, fortunately few, when sympathetic ophthalmia in its own inexorable way was steadily destroying the sight of both eyes. Now they have at least a fighting chance, with this shield to prevent tissue reaction, and to protect delicate structures until the attack of inflammation is past.

*Surgical progress*

It has been stimulating to work in a period during which so many technical advances have taken place in ophthalmology. In practically every year, including the war years, some new idea in eye surgery has been introduced, and we have travelled far since Daviel in 1747 first successfully removed a cataract, and v[illegible] Graefe in 1857 first relieved acute glaucoma by iridectomy. These classical operations are still in use and, although modified in technical details, they have remained important items in the ophthalmic surgeon's repertoire. In 1928 another major event occurred in the history of eye surgery, when Jules Gonin first introduced ignipuncture of the globe to seal off the holes which are usually present in serous detachment of the retina. Then at last it became possible, in suitable cases, to treat successfully a condition hitherto regarded as hopeless. Numerous ideas for replacing detached retinae followed—the principle involved being the use of electrical or chemical applications to the sclera to stimulate adhesions between the choroid coat and the separated area of the retina. By these methods, about 50 to 60 per cent. of all cases of retinal detachment can be cured, and in selected groups the prognosis is much better. In the prevention and treatment of blindness, one is always hoping for some development which will enable one to tackle successfully the type of case which ordinarily does badly, and in the case of detached retinae this has been found recently in the revival of an old idea which has become feasible owing to improved surgical technique. Many cases of detachment do not respond favourably to diathermy operations because for some reason, e.g. shrinkage of the vitreous, the retina does not come near enough to the area of choroidal coagulation to which it is intended to adhere. In such cases, and in others with a bad prognosis (e.g. high myopia, eyes in which the lens has been removed or congenitally dislocated, and detachments in old people), the operation of scleral resection has proved successful in an encouraging proportion of patients. The aim is to shorten the globe and thus bring choroid nearer retina, by removing a crescentic strip of sclerotic (full thickness, or all but a very thin lamella of scleral fibres) 4 mm. in width at its centre, and 10 to 14 mm. from the corneal margin. Up to half the circumference of the globe may be resected at one operation. The exposed choroid is painted with 1·5 per cent. to 3 per cent. caustic potash to stimulate adhesion to the retina, the sub-retinal fluid is drained off, and very fine silk sutures, previously inserted at the cut edges of the sclera, are tightened and tied to close the wound. A number of unpromising cases of detachment have been treated with good results in Bristol recently, the method used being lamellar sclerectomy either as a primary operation or following diathermy.

The history of corneal grafting, or keratoplasty, goes back to the early years of last century, but very few successes were recorded in the hundred years which followed. Members of the Society in 1936 had the pleasure of hearing Tudor Thomas, this year's President of the British Medical Association, deliver a lecture on the subject of corneal transplantation, of his own pioneer work on rabbits, and of his successful application of the operation to man in 1930 and subsequently. In the past twenty years, a large number of successful grafting operations have been performed by ophthalmic surgeons throughout the world, and many individual ideas in technique have been devised. Since the passing of the Corneal Grafting Act last year, donor material is becoming available, and corneal transplantation will in future appear more frequently on ophthalmic operation lists than has hitherto been the case. The idea of corneal grafting has made a considerable appeal to the laity, and on more than one occasion I have received touching letters from members of the public, offering one of their own sound eyes to restore the sight of a mother or other near relative. They naturally have little conception as to the limitations of the operation, and expect it to work wonders in all manner of unsuitable eye conditions. Not infrequently, although a clear graft may be obtained, the eye is subsequently found to be unsound in other respects, and the final visual result may not be so good as was hoped. Nevertheless, in a limited number of suitable cases, keratoplasty is likely to be retained as one of our most valuable surgical procedures. It is, of course, possible that there may be fewer opaque corneae requiring surgical treatment in future, owing to the beneficial effects of the sulphonamides, antibiotics and cortisone in reducing the severity of ocular infections.

Cataract surgery has developed considerably in the period under consideration. The older operation, in which the anterior capsule of the lens was opened or partly removed and the hard nucleus expressed, is still in use, although irrigation of the anterior chamber with warm saline is now frequently employed to remove residual cortical debris. The intra-capsular operation, or total extraction of the lens in its capsule, has been steadily gaining favour, however, especially for immature cataract. The technique is more difficult, but the operation has advantages over the other of freedom from post-operative iritis and from any necessity of needling a thickened posterior capsule.

Removal of the crystalline lens, by either method, however, from an eye previously in a normal optical state, renders the eye highly hypermetropic. Before useful sight can be obtained, a heavy and rather unsightly convex spectacle lens is required, in the wearing of which there are some optical disadvantages. The vision is usually clear, but the retinal image is magnified by about one-third, and confusion may also be caused by the prismatic effect of looking through the lens obliquely. Those who have had a cataract removed from one eye, the other having clear natural vision, find they can not see binocularly and may suffer discomfort from the inequality of visual acuity. These difficulties after a successful operation by either of the well-established methods are, of course, minor matters compared with the great benefit gained by cataract patients in recovering useful sight, even although the optical accessories required are rather clumsy. For generations, people have managed reasonably well after cataract extraction by using spectacles, and this is how the majority in future will continue to compensate for the lack of the crystalline lens. The difficulties have been minimized to some extent and in various ways—for example, by lenses whic are lighter, thinner and of special curvature, and by contact lenses. The latte especially in young patients after a monocular cataract has been removed, ha been used with success.in a small number of cases, but neat fingers are necessa for their insertion and removal, so that few aged patients are likely to succe in wearing them.

This brings me to what may prove to be one of the major developments i eye surgery of this century so far. At the Oxford Ophthalmological Congre in 1951, Harold Ridley first described an operation he had devised for replaci the cataractous lens by an acrylic lenticulus of appropriate strength. As preliminary to the insertion of the plastic lens, the cataract is removed by t older method of capsulectomy, expression of the nucleus, and washing out t residual sticky cortex. Only the posterior portion of the capsule then remai intact, and the acrylic lens is placed in front of that, and behind the iris oppos the pupil, thus restoring the normal optical properties of the eye, except acco modation. At first, the cases selected for operation were middle-aged peop with monocular cataract, and the main object was to restore binocular visio Subsequently, older patients were treated similarly in order to spare them t necessity of wearing heavy lenses for distance, and by now an impressive to of successful results has accumulated. Although the convalescence after t operation is a little prolonged, usually owing to slight reaction to the forei body by the iris, no serious complications have ensued which could be direc attributable to the presence of the acrylic lens if correctly inserted, by the autho technique, in an eye suitable for the procedure. This means an eye in whi the cataract is quite mature, or from which the remaining soft lens matter c be thoroughly washed out after removal of the nucleus. It excludes eyes wi very immature cataracts, for which the best procedure is total extraction in t capsule, and some others in which post-operative risks are considerable. Neve theless, this new operation is a notable advance within its own scope, and t majority of over a score of patients on whom my colleagues and I have by no carried it out at the Eye Hospital are well satisfied with the results.

Glaucoma, on the other hand, remains the most obstinate of eye problem It is not one condition, but several; it may be acute and congestive, chron and insidious, it may accompany what is generally considered normal or lo intraocular tension, or it may be secondary to other diseases of the eye. Th diagnosis may be easy, or so difficult that repeated examinations and provocati tests may still leave the matter in doubt. It is probably true to say that, althou intensive research has cast much light on the mechanism, biochemistry an pathology of the various types, no really outstanding development has take place in the actual treatment of the disease during the time I have been in pra tice. New miotics have been introduced, and the fashion in some quarters ha changed from trephining to iris inclusion operations, but glaucoma in all it varieties is still amongst the most difficult of eye diseases.

### THE FUTURE

To conclude this general survey, you might properly expect me to offer som constructive suggestions about ways of reducing the numbers of the blin There is obviously a need to continue research into the general medical an ophthalmological diseases which lead to loss of sight. The Institute of Ophthalmology, in the five years since its foundation, has gained an international reputation for advanced work in the biochemistry, biophysics and pathology of the eye. The Director of Research at the Institute is Sir Stewart Duke-Elder, who has himself made such notable contributions to the literature of ophthalmology, and especially to the study of glaucoma. He has on his staff a group of scientists who have already produced an impressive amount of original work. The exceptionally important and recently published communication by Ashton, Ward and Serpell (1953) on "The Role of Oxygen in the Genesis of Retrolental Fibroplasia" is a good example of its high quality. We can take it then that, both here and in centres of research abroad, a wide range of scientific investigation is proceeding. Pending further developments, those of us who are engaged on clinical ophthalmology must concentrate mainly on the treatment of cataract and glaucoma if we are to make any substantial contribution to the reduction of blindness. Sorsby estimated that about 20,000 people in this country were blind from cataract in 1950, and that this number "could be reduced by at least a third if existing methods of treatment were utilized to the full". Of the 11,000 additions made to the National Register, in the last year for which figures are available, I understand that about 3,000 were blind on account of cataract.

As a result of personal experience in examining many applicants for registration, my ideas about improving matters have become to some degree crystallized, and I put forward the following for consideration:

1. The certificate of blindness should be completed by a practising ophthalmologist only, and preferably of consultant rank. Answers are required to such questions as the possibility of treatment and the chance of blindness being cured; it is obvious that an ophthalmologist familiar with the potentialities of modern surgical and medical eye treatment is the only one who can give a reliable and useful opinion. As things are at present, it is quite possible that there are fewer recommendations for cataract and other operations than should be the case.

2. The idea that the patient has to go almost blind, and that the cataract has to be "ripe" before operation can be carried out, is widespread amongst the laity. Actually, the crystalline lens can be removed from the eye of a middle-aged or elderly person by the intra-capsular method, however early the stage of cataract. Operation is justifiable as soon as the eye affected is substantially handicapped by failing vision, and should take place as early as possible, while the patient is still in reasonably good general condition, otherwise the chance of doing it may slip past.

3. Patients' fears about "having to lie in a dark room with both eyes covered up for weeks" should be dispelled, and it should be explained to them that the operation is a brief one, entirely painless, and that both eyes are covered for two days only. The ward or room is shaded, not dark, and the patient is usually up in a week.

4. Out-patients under observation for cataract, glaucoma and other chronic ailments, who fail to keep up their periodical attendances, should be followed up by letter, and a home visit by a Welfare Officer, if necessary. Again and again, I have found at the examination of the blind that they had drifted into a sightless condition through becoming hospital absentees. In some cases it was due to difficulties about getting to the clinic, in others to apathy or lack of understanding. A Welfare Officer for the Prevention of Blindness was appointed in connection with the City Council's social services in 1945, and she is doing very useful work, not only by securing attendance of patients whose sight is in danger but also by encouraging them to carry out the advice given in hospital.

5. Glaucoma has been reckoned to be responsible for at least 10,000 cases of blindness in this country; it is much more difficult to deal with than cataract owing to problems of diagnosis and treatment. One thing certain is that determined effort should be made to detect the chronic type of the disease in its early stages. Far too many patients first come under the care of an ophthalmologist when gross defects in the fields and even in the central vision have already taken place. It is generally agreed that propaganda addressed to the public on this subject is neither likely to be effective, nor even desirable. The proper approach would be through members of the medical profession who are in contact with large numbers of the population, i.e. general practitioners and industrial health officers. In the United States, the method suggested for screening the population is that of measuring the intra-ocular tension by a simple and inexpensive tonometer, and a crusade is already on foot there, where it is estimated that about one million adults have glaucoma and do not know it. Since the existence of a scotoma near the fixation point is one of the earliest signs of the disease, Williamson-Noble (1953) considers that a simple device for detecting this would be preferable to the tonometer, and he has devised such an instrument. It is hoped that this will be given an extensive trial, for scotoma detection carried out on large numbers of the population might ultimately make a valuable contribution towards the prevention of blindness. Suspected cases would require further investigation, and this could be carried out at special glaucoma clinics attached to the larger eye centres of the clinical areas.

Although several items have been omitted, it is time to bring this balance sheet to a close—on the whole, as I hope will be agreed, a record of substantial progress, with a few disappointments, but a satisfactory reserve of enthusiasm and drive for further efforts. And this will be needed, for it would seem that, so long as the expectation of life increases, and the unexpended portion of intra-uterine life is passed (by the prematurely born) in the supercharged atmosphere of the oxygen tent, those of us who deal with the special senses can see no end to our struggle against the visual and other anomalies which at present inevitably arise. One day, perhaps, when the obstetrician and paediatrician have solved the riddle of prematurity, the physician and gerontologist the problem of reaching old age without senility, we ophthalmologists may be able to write off as settled a few more outstanding accounts.

### REFERENCES

Ashton, N., Ward, B., and Serpell, G. (1953). *Brit. J. Ophthal.*, **37**, 513.
Brown, C. A., and Corner, B. (1952). *Ibid.*, **36**, 281.
Sorsby, A. (1950). *Medical Research Council Memorandum* No. 24.
Sorsby, A. (1953). London: H.M. Stationery Office.
Thomas, J. W. T. (1936). *Bristol Medico-Chirurgical Journal*, **53**, 75.
Williamson-Noble, F. A. (1953). *Brit. J. Ophthal.*, **37**, 565.

